# Public access to protocols of contemporary cancer randomized clinical trials

**DOI:** 10.1186/s13063-021-05382-7

**Published:** 2021-06-27

**Authors:** Christopher Babu, Loren Mell, Nancy Lee, Kaveh Zakeri

**Affiliations:** 1 Weill Cornell Medical College, New York, NY USA; 2 Department of Radiation Medicine and Applied Sciences, University of California San Diego, La Jolla, CA USA; 3 Department of Radiation Oncology, Memorial Sloan Kettering Cancer Center, New York, NY USA

**Keywords:** Protocols, Clinical trials, Access, Cancer

## Abstract

Access to randomized clinical trial (RCT) protocols is necessary for the interpretation and reproducibility of the study results, but protocol availability has been lacking. We determined the prevalence of protocol availability for all published cancer RCTs in January 2020. We found that only 36.1% (48/133) of RCTs had an accessible protocol and only 11.3% of RCTs (15/133) had a publicly accessible protocol that was not behind a paywall. Only 18.0% (24/133) of RCTs were published in conjunction with the protocol on the journal website. In conclusion, few cancer RCTs have an accessible research protocol. Journals should require publication of RCT protocols along with manuscripts to improve research transparency.

**Supplementary Information:**

The online version contains supplementary material available at 10.1186/s13063-021-05382-7.

Randomized clinical trials (RCTs) are the gold standard for evaluating medical interventions, yet RCTs have been plagued by selective reporting and “spin” (i.e., misrepresentation of results) [1, 2]. Access to RCT protocols can serve as a public safeguard against biased clinical trial design and reporting, but protocol transparency has generally been lacking [3, 4]. Increasingly, some medical journals will publish standalone protocols for open access at the outset of a clinical trial [5]. However, protocol modifications are common, including changes to the eligibility, treatment, and primary endpoint [6], and thus, the final version of the amended protocol is needed for readers to fully interpret the scientific rigor and results of a study. Medical journals can play a critical role in improving the transparency of RCTs by requiring publication of all iterations of the protocol alongside trial manuscripts. While some high-impact medical journals require publication of protocols [7], less is known about the public availability of protocols for cancer RCTs published across the medical literature.

## Methods

Our primary aim was to determine the availability of research protocols in a contemporary cross-section of published cancer RCTs. We conducted a PubMed search of all published cancer RCTs in the month of January 2020. The search query (Additional file 1) yielded 1098 results that were assessed by two authors (CB, KZ) to determine if they were RCTs. For published RCTs that did not include a protocol in the online materials, we conducted an internet search including ClinicalTrials.gov, PubMed, and Google to determine whether a current or prior version of the protocol was available (Additional file 2). Only primary analyses of RCTs were included. Pilot RCTs and studies not written in English were excluded. Two-sided Mann-Whitney U and chi-square tests were used to compare differences between groups and the analysis was conducted in R.

## Results

**Fig. 1:**
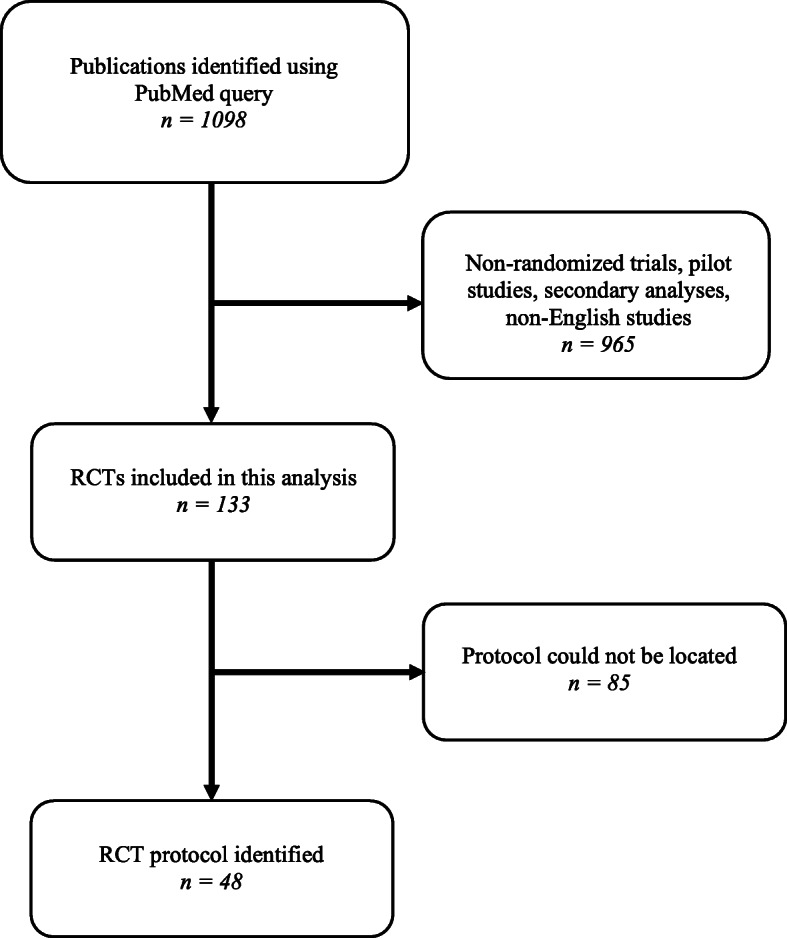
Study Flowchart. RCT, Randomized Clinical Trial

A total of 133 RCTs were included in the final analysis (Fig. 1). Within this cohort, the median study sample size was 128 and most studies investigated cancer-directed therapy (40.6%) or supportive care interventions (45.9%), such as symptom control, patient satisfaction, decision-making, and health literacy (Table 1). The most common primary endpoints included symptom management (29.4%), event-free survival (21.0%), and overall survival (9.1%). Notably, 4.5% of RCTs did not specify a primary endpoint, which is consistent with a prior systematic review of cancer RCTs [8].

**Table 1:** Study characteristics for randomized cancer clinical trials

	RCTs with protocols, n=48	RCTs without protocols, n=85
Sample size, median (range)	203 (7–13,195)	102 (6–3864)
Type of cancer, n (%)		
Central nervous system	0	3 (3.5)
Head and neck	3 (6.2)	5 (5.9)
Gastrointestinal	7 (14.6)	21 (24.7)
Lung	6 (12.5)	6 (7.1)
Genitourinary	11 (22.9)	13 (15.3)
Breast	9 (18.8)	20 (23.5)
Leukemia/lymphoma	5 (10.4)	6 (7.1)
Melanoma	1 (2.1)	2 (2.3)
Soft tissue sarcoma	1 (2.1)	1 (1.2)
Thyroid	0	1 (1.2)
Multiple	5 (10.4)	7 (8.2)
Study type, n(%)		
Cancer-directed therapy	25 (52.1)	29 (34.1)
Supportive care	17 (35.4)	44 (51.8)
Imaging	1 (2.1)	2 (2.4)
Preventative/screening	2 (4.2)	6 (7.0)
Surgical/anesthesia	1 (2.1)	4 (4.7)
Other	2 (4.2)	0
Primary endpoint^a^, n(%)		
Overall survival	4 (7.4)	9 (10.1)
Event-free survival	15 (27.8)	15 (16.9)
Response rate	4 (7.4)	6 (6.7)
Symptom management	14 (25.9)	28 (31.5)
Other	17 (31.5)	25 (28.1)
Not specified	0	6 (6.7)
Single primary endpoint	42 (87.5)	75 (88.2)
Co-primary endpoints	6 (12.5)	4 (4.7)
Trial phase, n (%)		
III	21 (43.8)	18 (21.2)
II	10 (20.8)	26 (30.6)
Not specified	17 (35.4)	41 (48.2)
Source of funding		
Industry	14 (29.2)	18 (21.2)
Academic/public	27 (56.2)	53 (62.3)
None listed	7 (14.6)	14 (16.5)

^a^Co-primary endpoints were counted twice

Most RCTs were supported by academic or public institutions (60.1%), followed by industry-sponsored RCTs (24.1%) and those without a stated funding source (15.8%).

In total, 48 RCT protocols (36.1%) were identified and only 24 protocols (18.0%) were published in conjunction with the RCT manuscript. Twelve protocols (9.0%) were previously published, 5 protocols (3.8%) were accessible at ClinicalTrials.gov, and 7 protocols (5.3%) were available elsewhere online. A total of 15 protocols (11.3%) were publicly accessible without a paywall. Of the RCTs with previously published protocols, only one included a protocol update with the published results. Phase III RCTs were more likely to have an identifiable protocol compared to other RCTs (Table 1; p=0.006). The median impact factor was significantly higher among journals that published protocols in conjunction with the RCT manuscript compared to journals that did not (7.0 vs 3.5; p< 0.0001). The median sample size among RCTs with an identifiable protocol was nearly double that of RCTs in which a protocol could not be found (203 vs 102; p=0.001). Median sample sizes were similar among RCTs published in conjunction with the protocol compared to those that were not (312 vs 184; p=0.56). There was no difference in protocol availability between industry sponsored and academic or publicly sponsored RCTs (43.8% vs 33.8%; p=0.32).

## Discussion

In summary, we found only a very small number of RCTs were published along with the protocol with only one published manuscript that included a protocol update. Journals with a higher impact factor were more likely to include RCT protocols. Access to RCT protocols is critical for transparency, reproducibility, and interpretation of the study results. More journals should require publication of RCT protocols in conjunction with the study results.

## Supplementary Information

**Additional file 1.** PubMed Search Query. Description: This additional file contains the complete original PubMed search query used to generate the initial study cohort of 1098 results, which were then reviewed to identify randomized clinical trials.

**Additional file 2.** Journal and Protocol Availability Status for All Included Trials. Description: This additional file contains the PubMed ID, journal and protocol availability status for all 133 studies included in this analysis.

## Data Availability

The datasets used and analyzed during the current study are available from the corresponding author on reasonable request. Additional file 1 contains the complete PubMed search query used to generate the initial study dataset. Additional file 2 contains the PubMed ID, journal, and protocol availability for the included randomized clinical trials.

## Abbreviations

RCT: Randomized clinical trial
