# Supplementary material for: Public access to protocols of contemporary cancer randomized clinical trials
Source: Trials. 2021 Jun 27;22:418. doi: 10.1186/s13063-021-05382-7 (PMC8237482; doi:10.1186/s13063-021-05382-7)
Supplement: Supplementary file 1 — Additional file 1. PubMed Search Query. Description: This additional file contains the complete original PubMed search query used to generate the initial study cohort of 1098 results, which were then reviewed to identify randomized clinical trials. [file 13063_2021_5382_MOESM1_ESM.pdf]

Additional File 1

PubMed Search Query:

(cancer[Title] OR leukemia[Title] OR lymphoma[Title] OR carcinoma[Title] OR melanoma[Title] OR sarcoma[Title] OR tumor[Title] OR tumour[Title] OR glioma[Title] OR glioblastoma[Title] OR astrocytoma[Title] OR oligodendroglioma[Title] OR brain metastases[Title] OR adenocarcinoma[Title] OR metastases[Title]) AND (Phase III[Title/abstract] OR Phase 3[Title/abstract] OR clinical trial[Title/abstract] OR trial[Title/abstract] OR randomized[Title/abstract] OR randomized[Title/abstract] OR randomised[Title/abstract] OR randomly[Title/abstract]) AND (("2020/01/01"[Date - Publication] : "2020/01/31"[Date - Publication]))
