# Supplementary material for: Public access to protocols of contemporary cancer randomized clinical trials
Source: Trials. 2021 Jun 27;22:418. doi: 10.1186/s13063-021-05382-7 (PMC8237482; doi:10.1186/s13063-021-05382-7)
Supplement: Supplementary file 2 — Additional file 2. Journal and Protocol Availability Status for All Included Trials. Description: This additional file contains the PubMed ID, journal and protocol availability status for all 133 studies included in this analysis. [file 13063_2021_5382_MOESM2_ESM.pdf]

Additional File 2.
Journal and Protocol Availability Status for All Included Trials.

PMID	Journal	Protocol Available (Y or N)
31995683	New England Journal of Medicine	Y
31790344	Journal of Clinical Oncology	Y
31806543	Lancet Oncology	Y
31411950	Journal of Clinical Oncology	Y
31944221	JAMA Oncology	Y
31839281	Lancet	N
31980913	Cancer Immunology, Immunotherapy	N
31037378	Supportive Care in Cancer	N
31785463	European Journal of Cancer	N
31767473	Radiotherapy and Oncology	Y
31976786	Journal of Clinical Oncology	Y
31918233	European Journal of Cancer	Y
31901705	European Journal of Cancer	Y
31995688	New England Journal of Medicine	Y
31776726	Annals of Hematology	Y
31935026	JAMA	Y
31879945	British Journal of Haematology	Y
31924316	European Urology	N
31958446	CHEST	N
31756547	International Journal of Surgery	N
31830347	Oral Diseases	N
31810797	Lancet Oncology	Y
31936897	Cancers	Y
31001692	Supportive Care in Cancer	N
30998526	European Journal of Cancer Prevention	N
31418073	Supportive Care in Cancer	N
31950156	Neurosurgery	N
31571329	European Journal of Cancer Care	N
31804876	Journal of Clinical Oncology	Y
31794324	Journal of Clinical Oncology	Y
31605312	Breast Cancer Research and Treatment	N
31853892	Der Urologe	N

31921608	Avicenna Journal of Phytomedicine	N
31971403	Health Psychology	N
32002556	Neuro-Oncology	N
31706052	Bone	N
31985125	The Oncologist	N
31983177	Asian Pacific Journal of Cancer Prevention	N
31869461	British Journal of Surgery	N
31725196	Cancer Medicine	N
31954884	World Neurosurgery	N
31948296	Journal of Investigative Surgery	N
31940376	PloS One	N
31685429	Oral Surgery, Oral Medicine, Oral Pathology and Oral Radiology	N
31932108	Gynecologic Oncology	N
31526964	Nutrition	N
31496287	Nutrition and Cancer	N
31785830	Radiotherapy and Oncology	Y
31903689	Head and Neck	Y
31600414	Psychooncology	N
32003822	JAMA Network Open	Y
31769226	Cancer Medicine	N
32037284	Clinical Nutrition	Y
31781890	Archives of Gynecology and Obstetrics	N
31753727	Lancet Oncology	Y
31956347	Journal of Cancer	N
31701267	Supportive Care in Cancer	Y
32090630	Integrative Cancer Therapies	Y
31788995	Journal of Gynecologic Oncology	N
31785451	Oral Oncology	N
31093677	Journal of the National Cancer Institute	N
31659822	Psychooncology	Y
31968063	JAMA Surgery	Y
31385041	Surgery Today	N
31906751	Journal of Investigative Surgery	N
31908102	European Journal of Cancer Care	N

31728618	Journal of Cancer Research and Clinical Oncology	N
31381147	International Journal of Cancer	N
31097634	Haematologica	N
31666284	Cancer Epidemiology, Biomarkers & Prevention	N
31352797	Nutrition and Cancer	N
31924332	Gynecologic Oncology	N
31291710	Cancer Research and Treatment	Y
31912784	Annals of Oncology	Y
31189338	Psychology & Health	N
31961463	BJOG	N
31455085	American Journal of Health Promotion	N
31402072	Journal of European Society of Surgical Oncology and the British Journal of Surgical Oncology	Y
31805025	Cancer Nursing	N
31999946	Lancet Oncology	N
31997146	Journal of Cancer Education	N
31838009	Lancet Oncology	Y
31760314	European Journal of Cancer	N
31903600	British Journal of Surgery	N
31898309	International Journal of Behavioral Medicine	Y
31595627	Psychooncology	Y
31931269	European Journal of Cancer	N
31615934	Clinical Cancer Research	N
31838561	Cancer, Chemotherapy, and Pharmacology	N
31892574	Anticancer Research	N
31975316	Breast Cancer Research and Treatment	N
31962198	European Journal of Cancer	N
31982605	Journal of Pain and Symptom Management	N
31953483	Prostate Cancer & Prostatic Diseases	N
32001530	Haematologica	N
31627177	International Journal of Radiation Oncology	N

32013277	Official Journal of the Association of Pain Management Anesthesiologists	Y
31907662	Surgical Endoscopy: Ultrasound and Interventional Techniques	N
32007834	Growth Hormone & IGF Research	Y
31990759	American Journal of Clinical Oncology: Cancer Clinical Trials	N
30982092	Supportive Care in Cancer	Y
31993719	Osteoporosis International	Y
31593756	International Journal of Radiation Oncology, Biology, Physics	Y
31670173	International Journal of Nursing Studies	N
31605663	BJU International	N
31937614	Clinical Cancer Research	Y
31978815	The Breast	N
31806540	Lancet Oncology	Y
31999296	JAMA Oncology	Y
31907649	Supportive Care in Cancer	N
32055717	Clinical and Translational Radiation Oncology	Y
31173341	International Journal of Cancer	N
31235074	The American Journal of Surgery	N
31953725	Surgical Endoscopy: Ultrasound and Interventional Techniques	N
31570566	Clinical Cancer Research	N
31804894	Journal of Clinical Oncology	Y
32000167	American Journal of Clinical Oncology	N
31791552	Gynecologic Oncology	Y
31900761	Quality of Life Research	Y
31912800	Annals of Oncology	N
31838010	Lancet Oncology	Y
31953188	Gastrointestinal Endoscopy	N
31724334	Cancer Medicine	N
31903657	Head & Neck	N

31995035	Kardiologia polska	N
32089455	Clinical Colorectal Cancer	N
31726490	Thoracic Cancer	N
31968595	Nutrients	Y
31682542	Journal of Clinical Oncology	Y
31953696	Breast Cancer Research and Treatment	N
31955002	Lung Cancer	N
31812890	Lung Cancer	Y
